# The Recurrence Rates at Three Years for the Conservatively Managed UTUC Cases Using NBI-Assisted Flexible Ureteroscopy and Holmium Laser Vaporization

**DOI:** 10.3390/medicina60121911

**Published:** 2024-11-21

**Authors:** Petrisor Geavlete, Valentin Iordache, Razvan Multescu, Alexandra Paunescu, Cosmin Ene, Razvan Popescu, Catalin Bulai, Bogdan Geavlete

**Affiliations:** 1 Department of Urology, “St. John” Emergency Clinical Hospital, Vitan-Barzesti 13, District 4, 042122 Bucharest, Romania; geavlete@gmail.com (P.G.); catalin.bulai@umfcd.ro (C.B.); bogdan_geavlete@yahoo.com (B.G.); 2 Faculty of General Medicine, “Carol Davila” University of Medicine and Pharmacy, Dionisie Lupu Street 37, District 1, 030167 Bucharest, Romania; 3 Department of Pathology, “St. John” Emergency Clinical Hospital, Vitan-Barzesti 13, District 4, 042122 Bucharest, Romania; marialexandra21@yahoo.com; 4 Department of Urology, “Prof. Dr. Th. Burghele” Clinical Hospital, Panduri 20, District 5, 061344 Bucharest, Romania

**Keywords:** UTUC recurrence, flexible ureteroscopy, Holmium laser, NBI

## Abstract

*Background and Objectives*: This study aimed to evaluate the recurrence rates at three years for upper tract urothelial carcinoma (UTUC) cases managed conservatively, using Narrow Band Imaging (NBI)-assisted flexible ureteroscopy and Holmium laser vaporization. *Materials and Methods*: The study group included 61 patients who were diagnosed with NBI-assisted visualization with superficial pyelo-calyceal urothelial tumor lesions, treated conservatively by the flexible ureteroscopic approach and Holmium laser vaporization, also assisted by NBI. This was compared with a control group with the same number of cases, which underwent the same procedure, but without NBI technology. Recurrence rates, the rate of patients who underwent nephroureterectomy, and cancer-specific survival were compared. *Results*: The relapse rate at 1 year was 3.3% in the study group, and respectively 8.2% in the control group (*p* < 0.05). Depending on the histological characteristics, at 1 year the relapse rates in the study group were 1.8% in patients with low-grade tumors and 20% in those with high-grade tumors. At 3 years, the relapse rate was 11.5% in the study group versus 18% in the control group, (*p* < 0.05): 7.1% in patients with low-grade lesions and 40% in patients with high-grade lesions versus 21.4% in patients with low-grade lesions and 100% in patients with high-grade lesions (both arms with statistically significant differences, *p* < 0.05). Cancer-specific survival was 93.4% in the study group versus 86.9% in the control group (*p* < 0.05). *Conclusions*: The recurrence rates at three years for the UTUC cases managed conservatively, using NBI-assisted flexible ureteroscopy and Holmium laser vaporization, were lower than in patients treated by the same technique without NBI assistance, both in low- and high-grade tumors. Cancer-specific survival was also significantly improved by the association of NBI visualization during diagnosis and laser vaporization.

## 1. Introduction

Upper urinary tract tumors are not very frequent, but their incidence rate has risen as a result of improved detection and improved bladder cancer survival [1,2].

Patients who are treated using the conventional “gold standard” method of radical nephroureterectomy (RNU) are facing the consequences of losing an entire kidney unit and, potentially, a significant decrease in the glomerular filtration rate, which, in selected cases, may have a severe impact [3]. For this reason, conservative treatment alternatives were imagined and proposed in order to be applied to such patients. There is a large spectrum of kidney-sparing strategies aimed at preserving kidney function while effectively managing the cancer, such as segmental ureterectomy and endoscopic ablation (EA) techniques [4]. The allure of these conservative methods lies in their ability to achieve in selected cases comparable oncological outcomes to the traditional radical nephroureterectomy (RNU) while minimizing the associated morbidity and preserving more of the renal function. They are an option for patients with low-risk disease and selected patients with an imperative indication such as a solitary kidney or serious renal insufficiency [5,6,7,8,9,10], for whom kidney sparring treatment is the preferred approach according to the European Association of Urology (EAU) guidelines [11].

However, despite the advantages of kidney-sparing approaches, there are challenges to be addressed, particularly in the realm of diagnostic accuracy and consequently in the process of properly selecting these cases. A ureteroscopic biopsy is associated with a high risk of sampling error, with significant rates of under-grading and/or under-staging [12]; thus, this proportion of patients will be under-treated with EA. New technologies such as confocal laser microscopy [13] promised to solve this issue, but none of them have validated diagnostic criteria yet. Unfortunately, neither approach has been anchored in the current EAU guidelines to safely push the limits of kidney-sparing surgery [11].

In the last twenty years, due to the significant improvement of endoscopic and imaging techniques, there has been a paradigm shift in the management of UTUC toward a more personalized, risk-stratified approach [14,15].

This evolution has been facilitated by advancements in endoscopic and imaging techniques, which have allowed for more precise tumor characterization and treatment planning.

Initially, segmental ureteric resection, ureteroscopic resection, and percutaneous resection were proposed as such techniques.

Nowadays, the mainstay of endoscopic treatment is laser ablation with various devices such as Holmium:YAG (Ho:YAG) laser, which produces tissue ablation reaching an incision depth of 2 mm and coagulation thickness of 0.48 mm [16]. In contrast, the Thulium:YAG (Tm:YAG) laser produces larger coagulation areas [17] while the new super pulsed Thulium Fiber Laser (TFL) had a smaller coagulation area and incision depths than the Ho:YAG laser [18]. In our study we employed laser ablation as the method of choice for conservative treatment of UTUC.

Moreover, the integration of NBI into flexible ureterorenoscopy has revolutionized the management of UTUC, allowing for enhanced visualization of suspicious lesions and improved diagnostic accuracy as well as having the potential to optimize the conservative treatment itself. Specifically, NBI was confirmed as improving the detection of superficial bladder tumors [19], so it started to also be used for the detection of UTUC malignancies due to progress offered by digital flexible ureteroscopes incorporating the NBI technology within the same setup [20].

Against this backdrop, the present study aims to contribute to our understanding of UTUC conservative management by evaluating the long-term outcomes of NBI-assisted flexible ureteroscopy and Ho:YAG laser ablation of such tumors. With a better visualization of the extension of the tumoral tissue, we believe we are able to optimize its complete ablation. By assessing recurrence rates at three years post-intervention, we hope to shed light on the efficacy and durability of these strategies, ultimately informing clinical practice and optimizing patient care.

## 2. Materials and Methods

The current study represents a continuation of research efforts previously published in another journal [21], which focused on the nuanced detection and management of superficial pyelo-calyceal urothelial tumor lesions with the aid of NBI. Between 2015 and 2018, the initial prospective study enrolled consecutive patients with primary suspicion of non-invasive UTUC and focused on diagnosis improvement using NBI technology. The present study is a prospective extension of that evaluation, in which we focused on the next step applied to the patients diagnosed with pTa UTUC: conservative treatment enhanced by the visualization technique.

In this longitudinal investigation, a cohort comprising 61 patients diagnosed with such lesions underwent conservative treatment utilizing a flexible ureteroscopic approach and Ho:YAG laser vaporization, all while benefiting from NBI assistance.

To establish a meaningful basis for comparison, a control group consisting of 61 patients with anatomo-pathologically confirmed pyelo-calyceal urothelial tumors was meticulously paired with the study group. Unlike the study cohort, these patients underwent Ho:YAG laser vaporization using a flexible ureteroscope without the assistance of NBI during both diagnosis and treatment. The matching process ensured that the control group was comparable to the study group regarding anatomo-pathological characteristics and minimized potential confounding variables such as sex and age distribution.

**Figure 1:**
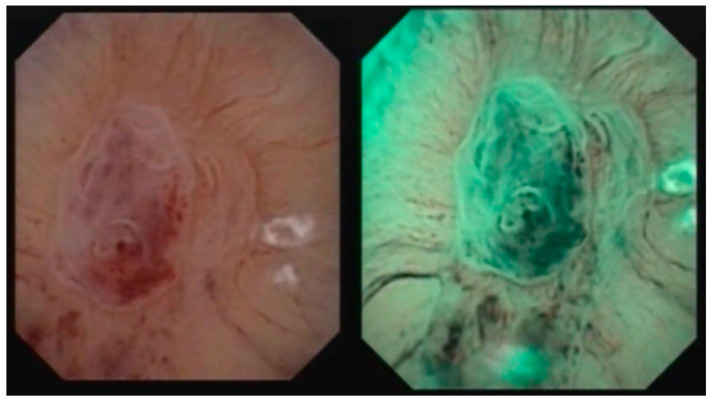
White light and NBI image of a pyelocaliceal tumor.

**Figure 2:**
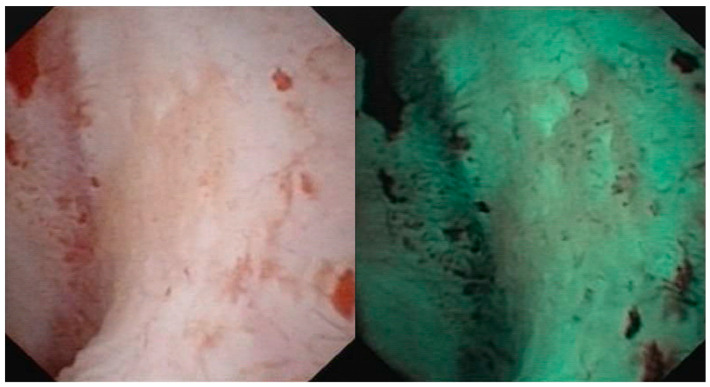
White light and NBI image of a pyelocaliceal tumor.

The protocol for the selection of the study group patients comprised a contrast CT scan, urinary cytology, abdominal ultrasonography, cystoscopy, and digital flexible ureteroscopy. The endoscopic inspection of the entire pyelo-caliceal system (renal pelvis and all calyces) was performed in both white light and NBI and the suspected tumoral lesions were marked on a map. After the map of suspicious areas was created, all the lesions visualized in any of the two modes (either white light or NBI (Figure 1 and Figure 2)) were biopsied. For this stage, a biopsy (grasping) forceps was used in all cases. When performing this stage, special attention was given to going as deep as possible in the pyelo-caliceal wall, in order to obtain good samples, with minimal staging error.

**Figure 3:**
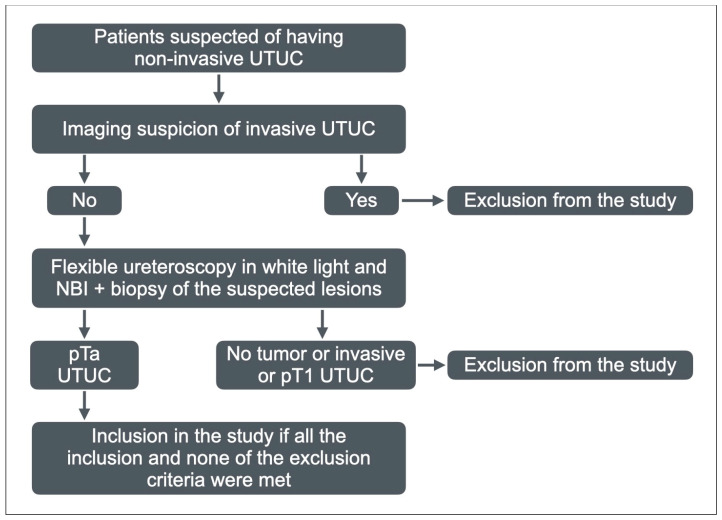
Selection process of the patients to be included in the study group.

This diagnostic protocol was applied in 87 consecutive patients admitted for primary suspicion of non-invasive UTUC (those with imaging signs of invasive tumors being excluded for the beginning). After the pathological exam, patients with invasive tumors and those with invasion in the lamina propria were also excluded. Finally, we identified 61 patients with malignant tumors that met all the inclusion criteria and did not have any exclusion criteria, and included them in the study group (Figure 3).

The inclusion criteria for both the study and control groups were carefully delineated to ensure the consistency and validity of the findings. Participants were required to be over 18 years old, possess a life expectancy exceeding three years, and be exclusively diagnosed with pTa urothelial carcinoma lesions at the pyelo-calyceal level. Exclusion criteria were put in place to mitigate potential biases, such as the presence of tumors larger than 2 cm or individuals unable to commit to a minimum three-year follow-up.

As recommended by the guidelines [11], the use of conservative treatment in all the cases was discussed as a shared-decision making process with the patient. The possibility of nephroureterectomy as the radical treatment was thoroughly discussed and its indication, importance, and impact were emphasized, especially in the patients with high-grade tumors (high-risk patients) and their choice was carefully recorded in the informed consent documents. All this process was repeated at all stages during the study, being emphasized to all the patients that, irrespective of the study design, their decision could reversed at any time. Details regarding the presence of imperative indications can be found in the Section 3.

Following the detection stage, we continued with the actual treatment procedure, employing a digital flexible ureteroscope with NBI capabilities and a 35 W Holmium laser, with 275-micron fiber. We performed laser vaporization of all the identified lesions (both in white light and NBI). After the complete destruction of the tumoral tissue apparent in white light, we switched to NBI to ensure that no tumors or tumoral margins with suspicious aspects remained. If such lesions were identified in NBI, laser ablation was performed as well, until complete removal of the macroscopically visible tumor was achieved. The laser settings were 0.6 J and 15 Hz, as comprised in the “Soft tissue” mode of the Ho:YAG laser machine. At the end of the procedure, another inspection of the renal pelvis and all the calyces was performed in both white light and NBI, in order to minimize the risk that tumoral lesions remained untreated.

Within the study group, the distribution of patients based on tumor grade was noted, with 56 presenting low-grade pTa tumors and 5 exhibiting high-grade pTa tumors. The control group mirrored this distribution, further enhancing comparability between the two cohorts.

Following the initial intervention, a comprehensive assessment was conducted at the 10-week mark post-treatment to identify and address any residual tumor tissue.

Subsequent evaluations at 1 and 3 years allowed for a detailed comparison of outcomes, including recurrence rates, the incidence of patients necessitating nephroureterectomy, and cancer-specific survival between the study and control groups. We included in the analysis only upper urinary tract recurrences (and no bladder ones) in order to avoid a potential bias source. This decision was imposed by the fact that we evaluated the impact of NBI visualization during laser ablation (in other words the impact of the completeness of conservative ablation of the tumors), while the bladder recurrence would have been probably consequential to other factors during the natural evolution of such cases.

Concerning the specimen handlings after biopsy, it should noted that cold cup forceps, diathermy forceps, or a tiny diathermy loop [22] can be used to take biopsies. In all our cases, we collected the biopsy specimen using the cold cup grasping forceps, in order to avoid artifacts during the pathological exam [23], followed by cauterization of the urothelial defect using a Bugbee electrode, in order to ensure a good quality hemostasis. Complete paraffin embedding of the biopsy specimens was required for histological analysis. The biopsy specimens may reveal velvet, erythematous, or papillary neoplasms in the urothelium, which may indicate tumor or inflammation [24,25]. These biopsies were then placed in different jars and paraffin-embedded in various blocks. For each biopsy, tissue slices at least three distinct levels are required for a histological evaluation [24,25]. Once a tumor has been discovered, the sample size should be appropriate for its size (at least one section should be collected for every centimeter of tumor). It should enable evaluation of the grade, histological type, and depth of penetration of the tumor.

To analyze the collected data rigorously, statistical methods, including Student’s t-test and chi-square test, were employed utilizing the Statistical Package for the Social Sciences (SPSS, IBM Corp, Armonk, NY, USA) version 20.0. This meticulous statistical approach aimed to uncover any significant differences between the two groups regarding outcomes and survival rates, thereby providing valuable insights into the effectiveness of the employed treatment modalities.

The study was conducted in accordance with the Declaration of Helsinki, the protocol was approved by the Ethics Committee and all the patients signed an informed consent form accordingly.

## 3. Results

Although sometimes the approach of certain areas of the pyelo-caliceal system may prove impossible due to various factors (stenosis of calyces, complex upper urinary tract architecture), in our study, complete access to all calyces was achieved in all patients.

Most of the included patients (106 out of the 122 patients) presented imperative indications for conservative treatment: solitary kidney, severe chronic kidney disease, and severe co-morbidities impeding radical treatment. All patients with detected high-risk diseases had such imperative criteria. They were also offered nephroureterectomy as a treatment choice, emphasizing the higher risk of progression and the potential impact over survival in the case of choosing the conservative methods. All these patients refused radical treatment, and the ureteroscopic laser ablation was applied in their cases after a case-by-case analysis.

At 10 weeks, residual tumor tissue was identified by a flexible ureteroscopic approach under white light in 8.2% (5 patients) of the study group, and 34.4% (21 patients) of the control group (*p* < 0.005). In all these patients, the remaining tumor tissue was re-biopsied and removed by laser ablation. White light inspection was applied to patients in both groups in order not to introduce sources of error into the statistical study. The relapse rate at 1 year was 3.3% in the study group, and respectively 8.2% in the control group (*p* < 0.05). Depending on the histological characteristics, at 1 year the upper urinary tract recurrence rates in the study group were 1.8% in patients with low-grade tumors and 20% in those with high-grade tumors. In the control group, these rates were 7.1% (statistically significant difference, *p* < 0.05), and respectively 20% (statistically insignificant).

At 1 year, two patients in the study group underwent nephroureterectomy, compared to three patients in the control group (statistically insignificant difference, *p* = 0.16).

**Figure 4:**
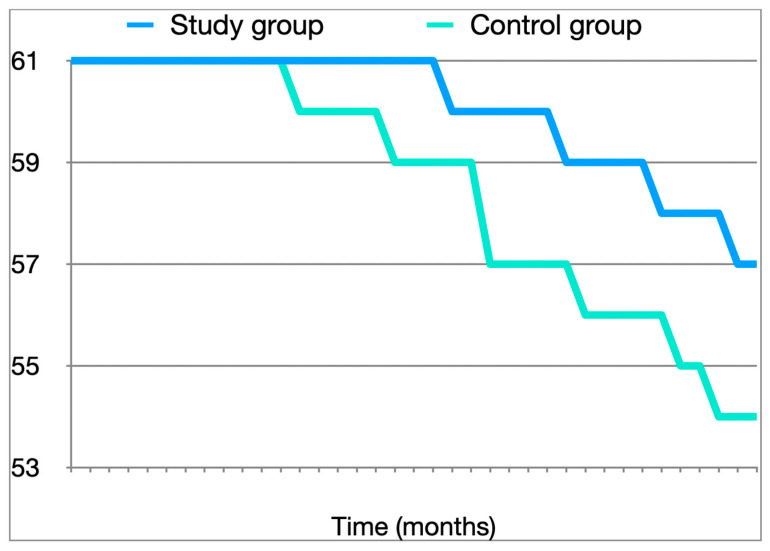
Kaplan–Meier curves for CSS.

At 3 years, the upper urinary tract recurrence rate was 11.5% in the study group versus 18% in the control group, (*p* < 0.05): 7.1% in patients with low-grade lesions and 40% in patients with high-grade lesions versus 21.4% in patients with low-grade lesions and 100% in patients with high-grade lesions (both arms with statistically significant differences, *p* < 0.05 (Table 1). At 3 years, four patients in the study group required nephroureterectomy compared to six patients in the control group (Table 2). Cancer-specific survival was 93.4% in the study group versus 86.9% in the control group (*p* < 0.05) (Figure 4). The site of recurrence in the two groups is described in Table 3.

## 4. Discussion

EA techniques are associated with a high risk of under-grading and under-staging, which makes the definition of a low-risk tumor unreliable and consequently could result in a high recurrence rate [4]. Furthermore, there are several other issues associated with EA that patients need to consider. Cancer-specific survival and overall survival might be comparable in cases for which the indication for kidney-sparing surgery is evidence-based after a benefit-risk evaluation [15]. However, the risk of recurrence after EA is up to 40% [26], comparable to the rate for non-muscle-invasive bladder cancer. Other studies showed a 5-year intravesical recurrence-free rate of 41.5% after surgery [27]. In our study, the recurrence rate at one year was similar (20%), while at three years it was 40% versus 100% for the high-risk tumors in the study group versus the control group; the values being similar to those from the studies mentioned above.

NBI is a visualization mode used during endoscopic evaluation, in which light is filtrated in order to select wavelengths of 415 nm (blue) and 540 nm (green). At these wavelengths, the absorption of light by hemoglobin is at the peak level so the blood vessels will appear dark and, consequently, will be more visible and the contrast of capillaries will be significantly better [28]. This property can be used to increase the identification of various urothelial lesions (and especially tumoral ones). After the initial benefits demonstrated for diagnosis of urothelial tumors in the bladder and after the inclusion of NBI capabilities in flexible digital ureteroscopes, the method was applied also for the urothelium in the upper urinary tract. It was hypothesized that the benefits of better tumoral identification for diagnosis purposes can also be extrapolated during treatment procedures. As we also stated while defining the study aims, clear and complete visualization of the tumoral borders and sometimes satellite malignant lesions translates into a complete resection and consequently better oncological outcomes in the long run.

Conservative management of UTUC requires even more meticulous follow-up given the need for additional endoscopic monitoring of the upper urinary tract [15], but sometimes with excellent outcomes, as observed in our evaluation: relapse rates for low-grade tumors patients in the study group were only 1.7% at one year and 7.1% at three years, versus 7.1% at one year and 21.4% at three years in the control group.

Within 6–8 weeks following the initial conservative flexible ureteroscopic intervention, a second-look endoscopic treatment is advised by EAU recommendations. Furthermore, depending on the UTUC risk class, patients treated conservatively with repeated ureteroscopic approaches must undergo strict endoscopic follow-up [11]. Only Proietti et al. conducted a systematic second-look flexible ureteroscopy; in situations with non-complete ablation at the first operation, Defidio et al., Wen et al., and Musi et al. conducted early second procedures [17,29,30,31].

All in all, UTUC recurrence represents the main concern in conservative treatment. The recurrence rate is variable depending on the different studies, varying between 20 and 85% and being even greater the higher the tumor grade [32]. Having this in mind, the diagnosis must be precise for better results following a conservative approach. CT urography is the imaging technique with the highest accuracy for UTUC [10], but also, the use of NBI could represent a certain advantage in tumor detection improving visualization of UTUC and enabling diagnosis of lesions non-visible in white light, according to some authors [33].

Regarding high-risk UTUC, diagnostic ureteroscopic evaluation combined with tumor biopsy seems to be a valuable tool for a better selection of cases for curative surgery. In such cases, the method proved to have a reduced rate of negative biopsy rate and also demonstrated good concordance with final pathology reports, produced after radical treatment. This good correlation was especially evident in high-volume centers [34].

Concerning the energy source used in the conservative treatment, Ho:YAG laser is the most frequently used energy source, with the settings usually being 0.5–1 J for energy and 10–15 Hz for frequency, with a tissue penetration depth of 0.4 mm, as used in our study, but other studies also mentioned the use of Nd:YAG laser in addition to Ho:YAG due to its deeper penetration in the tissue of around 5–6 mm [35,36].

In the literature, five studies reported the indication for a radical nephroureterectomy in cases of conservative endoscopic UTUC therapy failure during follow-up. The range of these reports was from 3.7 to 39.7% of cases [17,29,30,31,37,38]. According to Hsieh et al. [37], two patients developed metastases, and four patients (12%) died of cancer-related causes during follow-up.

Regarding the use of Thulium lasers, the results in patients treated with such an energy source were compared to a control group of patients treated with nephroureterectomy in the study by Wen et al. [30]. The authors observed that patients in the Thulium laser group had a greater tumor recurrence rate of 21.9% versus 7.8% in the radical surgery group (*p* < 0.01), but presented a shorter time of hospitalization and lower postoperative creatinine level.

The effect of adjuvant therapies, such as chemotherapeutic agents and/or immunotherapy with BCG, after kidney-sparing surgery for papillary non-invasive (Ta-T1) UTUCs and of adjuvant BCG for the treatment of upper tract CIS, was examined in a systematic review and meta-analysis evaluating the oncologic outcomes of patients with papillary UTUC or CIS of the upper tract treated with adjuvant endocavitary treatment. The study found no differences in terms of recurrence, progression, cancer-specific survival, and overall survival amongst the different drug administration methods (antegrade, retrograde, and combined approach). Moreover, the rates of recurrence after adjuvant instillations are similar to those documented in the literature for individuals who did not receive treatment, casting doubt on their effectiveness [39].

The surveillance of UTUC patients, especially those with a history of bladder cancer, necessitates a vigilant approach. Regular follow-up examinations, including cystoscopy and urinary cytology, are essential for detecting local recurrence, metachronous bladder tumors (probability increases over time [27]), and distant metastasis, as the risk evolves following initial treatment. In this context, it was observed that, at the follow-up of the patients with non-muscle invasive bladder cancer treated with BCG, 13% had UTUC at one year [40], while after radical cystectomy after muscle-invasive bladder cancer, 3–5% of the patients developed UTUC [41,42]. That is why the surveillance protocol is very strict and includes cystoscopy and urinary cytology [27,43]. It was observed that the risk of recurrence and death evolves during the follow-up period after surgery [44] so, as a consequence, there is a direct relationship between event-free follow-up and survival probability after radical nephroureterectomy [45].

Radical nephroureterectomy remains the gold-standard treatment for high-risk UTUC, except for very selected cases of distal ureteral tumors, for which conservative techniques can be proposed [46]. Radical nephroureterectomy was also chosen in our study for the selected cases, whenever the oncological situation required that attitude, with significantly more cases converted in the control group than in the study group. Cancer-specific survival of 93.4% in the study group versus 86.9% at three years, was relatively similar with other studies from the literature for the same follow-up period and even for a larger interval [47].

It would be also important to mention that the study has a few limitations, because of the relatively reduced number of cases and because it is a single-center study. As mentioned before, this evaluation is an extension of a previously published detection study. While a power analysis was conducted for that series to determine the appropriate sample size and ensure that it had sufficient statistical power, this was not possible for the actual paper, as it was a continuation of evaluating some already existing cases. However, because we already had a significant number of patients (taking into account the incidence of the disease), we felt that it was an opportunity to extend it even in these conditions. Also, we included in the detection study and, consequently, in the current extension, consecutive cases, which aims to increase objectivity, but also, taking into consideration that we are a tertiary center with experience in conservative treatment of UTUC, may also paradoxically become a source of bias.

Last but not least, it is important to note that kidney-sparing care was found to be a more affordable option than radical nephroureterectomy, saving USD 252,272 on average per patient over five years [48]. We were unable to locate any research that examined the effects of Thulium laser in the context of UTUC conservative treatment from an economic perspective. In endoscopic laser lithotripsy, Ryan et al. recently reported that TFL has a much shorter operative time and lower cost when compared to the usual Ho:YAG [49]. Future research is required to determine the cost-effectiveness ratio of Thulium lasers in patients with UTUCs in comparison to radical nephroureterectomy or Ho:YAG.

## 5. Conclusions

In conclusion, the upper urinary tract recurrence rates at three years for the UTUC cases managed conservatively, using flexible ureteroscopy and NBI-assisted Holmium laser vaporization, were lower than in patients treated with the same technique but without the benefits of NBI visualization, both in low and high-grade tumors. Cancer-specific survival was also significantly improved by the association of NBI visualization during both diagnosis and laser vaporization.

## Figures and Tables

**Table 1:** The relapse rate in the study groups.

Follow-Up Period	Study Group		Control Group		Statistical Evidence	Statistical Evidence
	High Grade	Low Grade	High Grade	Low Grade	High Grade	Low Grade
1 year	20%	1.8%	20%	7.1%	*p* > 0.05	*p* < 0.05
3 years	40%	7.1%	100%	21.4%	*p* < 0.05	*p* < 0.05

**Table 2:** The number of patients who underwent nephroureterectomy.

Follow-Up Period	Study Group	Control Group	Statistical Evidence
1 year	2	3	*p* = 0.16
3 years	4	6	*p* < 0.05

**Table 3:** The site of recurrence in the study groups.

Follow-Up Period	Place of Recurrence	Number of Lesions	(Orthotopic/Heterotopic)
		Study Group	Control Group
1 year	Renal pelvis	1(0/1)	2(1/1)
	Inferior calyx	1(1/0)	3(3/0)
	Medium calyx	2(0/2)	2(1/1)
	Superior calyx	0(0/0)	4(3/1)
	Total	4(1/3)	11(8/3)
3 years	Renal pelvis	4(2/2)	5(2/3)
	Inferior calyx	2(0/2)	7(3/4)
	Medium calyx	5(1/4)	7(3/4)
	Superior calyx	4(2/2)	6(5/1)
	Total	15(5/10)	25(13/12)

## Data Availability

The original contributions presented in the study are included in the article, further inquiries can be directed to the corresponding authors.
